# ABO allele-level frequency estimation based on population-scale genotyping by next generation sequencing

**DOI:** 10.1186/s12864-016-2687-1

**Published:** 2016-05-20

**Authors:** Kathrin Lang, Ines Wagner, Bianca Schöne, Gerhard Schöfl, Kerstin Birkner, Jan A. Hofmann, Jürgen Sauter, Julia Pingel, Irina Böhme, Alexander H. Schmidt, Vinzenz Lange

**Affiliations:** DKMS Life Science Lab, Blasewitzer Str. 43, 01307 Dresden, Germany; DKMS, Kressbach 1, 72072 Tübingen, Germany

**Keywords:** Blood group, ABO, NGS, Genotyping, Illumina, Amplicon

## Abstract

**Background:**

The characterization of the ABO blood group status is vital for blood transfusion and solid organ transplantation. Several methods for the molecular characterization of the *ABO* gene, which encodes the alleles that give rise to the different ABO blood groups, have been described. However, the application of those methods has so far been restricted to selected samples and not been applied to population-scale analysis.

**Results:**

We describe a cost-effective method for high-throughput genotyping of the ABO system by next generation sequencing. Sample specific barcodes and sequencing adaptors are introduced during PCR, rendering the products suitable for direct sequencing on Illumina MiSeq or HiSeq instruments. Complete sequence coverage of exons 6 and 7 enables molecular discrimination of the ABO subgroups and many alleles. The workflow was applied to ABO genotype more than a million samples. We report the allele group frequencies calculated on a subset of more than 110,000 sampled individuals of German origin. Further we discuss the potential of the workflow for high resolution genotyping taking the observed allele group frequencies into account. Finally, sequence analysis revealed 287 distinct so far not described alleles of which the most abundant one was identified in 174 samples.

**Conclusions:**

The described workflow delivers high resolution ABO genotyping at low cost enabling population-scale molecular ABO characterization.

**Electronic supplementary material:**

The online version of this article (doi:10.1186/s12864-016-2687-1) contains supplementary material, which is available to authorized users.

## Background

ABO is the clinically most relevant blood group system in transfusion and transplantation medicine [1]. Using classical serological methods, donor/recipient pairs are routinely classified phenotypically into four major blood groups (A, B, O, and AB). Additional phenotypes with weak expression patterns are recognized and have been adopted for ABO subgroup classification [2].

To supplement serological typing, several medium- to high-throughput molecular typing methods have been developed for the glycosyltransferase encoding *ABO* gene on human chromosome 9. These methods rely on a broad range of techniques such as restriction fragment length polymorphism (RFLP), sequence-specific primer (SSP) PCR, single-strand conformation polymorphism (SSCP) analysis, or DNA microarray hybridization [3–6]. Most molecular typing methods exclusively target exons 6 and 7, which code for the catalytic domain and comprise the majority of the coding sequence, and focus on single-nucleotide polymorphisms within these two exons.

While these methods generally suffice for clinical applications [7], they do not easily scale to the requirements of routine upfront ABO genotyping of large cohorts of blood donors [4]. Moreover, the currently most commonly used molecular typing methods are restricted to detecting the specific set of alleles included in the assay. Novel alleles are unlikely to be detected. To date, 367 *ABO* alleles are reported in the Blood Group Antigen Gene Mutation Database (BGMUT, http://www.ncbi.nlm.nih.gov/projects/gv/rbc). 95 alleles have been added to BGMUT since the database was last described in an academic publication four years ago [8]. As this trend is not likely to slow down in the short term, DNA sequencing of the entire exonic sequences should be employed for more accurate genotyping results. Several studies have discussed the application of next generation sequencing (NGS) for ABO blood group genotyping or demonstrated its potential [9–12]. However, so far it has not been applied at population-scale to determine *ABO* allele frequencies.

Our lab has been applying NGS for high-throughput HLA genotyping based on direct sequencing of amplicons on the Illumina MiSeq and HiSeq platforms since beginning of 2013 [13]. We are providing this service to stem cell donor centers, mainly DKMS German Bone Marrow Donor Center and affiliated centers, to characterize newly registered potential stem cell donors. More than 2.5 million volunteers have been typed using our NGS approach since 2013. The implementation of the NGS-based workflow for HLA genotyping has reduced costs by more than 50 % as compared to Sanger-based genotyping. Furthermore, this type of workflow allows adding additional genes of interest to the donor genotyping profile at a minimal surcharge. Since donor-patient matching of the ABO status simplifies transplantation-related patient treatment procedures and may even improve outcome [14], we chose to extend the existing NGS-based HLA genotyping workflow to additionally provide ABO genotyping. Currently, the major bone marrow donor registries accept ABO data only at the blood group resolution level (A, B, AB, O). Therefore, we designed the assay to provide ABO blood group resolution at minimal costs. This resulted in the selection of exons 6 and 7 for sequencing, which, based on the currently known alleles, enable unambiguous determination of the ABO blood group status. Meanwhile we have typed 1.69 million samples using this approach.

Here, we describe the workflow and analyze the level of resolution beyond the blood group level that may be obtained by an exon 6 and 7 restricted approach: 99.9 % of the samples can be resolved at the ABO allele or allele group level. We analyze a subset of 113,367 samples of German descent to report *ABO* genotype frequencies at the resolution level of allele groups. Furthermore, our workflow readily identifies so far unknown alleles and we describe 287 unique novel *ABO* variants in exon 6 and 7.

## Results

### Workflow

We describe a high-throughput workflow for ABO genotyping based on direct PCR amplicon sequencing on Illumina MiSeq or HiSeq instruments as described for HLA typing in Lange et al. [13]. The main advantages of this workflow are simplicity and cost effectiveness. Hundreds of samples may be pooled immediately after the PCR reaction as the samples are tagged during PCR with a molecular barcode that is read during sequencing. This approach significantly reduces costs and hands-on time for all post-PCR processing steps. In addition, since adapters are incorporated during the PCR, only four straightforward steps are required to initiate sequencing: PCR cleanup, quantification, denaturation and dilution.

**Fig. 1:**
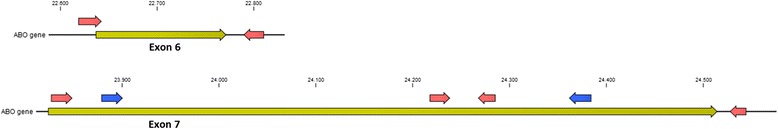
Primer locations for the *ABO* gene exons 6 and 7. Primers for ABO assay 1 are marked red. Primers for ABO assay 2 are identical to assay 1 in exon 6 and marked blue in exon 7

Amplicon length is restricted to the combined forward and reverse sequencing read length, currently 600 (MiSeq) or 500 (HiSeq) bases. We designed two assays targeting exons 6 and 7 of the *ABO* gene (Fig. 1, Additional file 1: Table S1). Since the length of exon 7 (686 to 692 bp) exceeds the amplicon size limit of our approach, ABO assay 1 includes two overlapping PCR reactions to cover exon 7 and one PCR reaction targeting exon 6 (Fig. 1). This delivers high resolution but prevents multiplexing the PCR in one reaction. ABO assay 2 is set up as multiplex PCR reaction covering exon 6 and the central 507 bases of exon 7 (Fig. 1). The multiplexed ABO assay 2 was developed to simplify the automated hitpicking setup.

The workflow has been applied routinely as part of the stem cell donor typing program since April 2014. As of March 2016, a total of 1.69 million samples have been successfully ABO genotyped. ABO assay 1 PCR is performed alongside assays for HLA, Rh, CCR5Δ32 and KIR genotyping using 48.48 or 192.24 Fluidigm Access Array chips or 384-well PCR plates. In addition, ABO assay 2 is applied on 384-well PCR plates to confirm results that do not meet the internal quality criteria or failed in the first round.

In our setting sequencing yields on average more than 1000 reads per amplicon and sample. These reads are matched to the alleles as listed in the BGMUT database [8] by our NGS genotyping software neXtype [13]. In contrast to many other molecular approaches our sequencing assays are not limited to detect a subset of the more frequent alleles but may rather detect the full spectrum of frequent and rare alleles as well as so far undescribed variants. Our restricted focus on exons 6 and 7, however, limits the obtainable resolution to alleles that differ in those exons. Seven alleles lacking exon 6 and 7 sequence information, four alleles lacking the major part of exon 7 sequence information, 19 alleles lacking phenotype information, and one allele of uncertain phenotype were excluded (Additional file 1: Table S2) reducing the data basis to 108 *A*, 68 *B* and 73 *O* alleles.

**Fig. 2:**
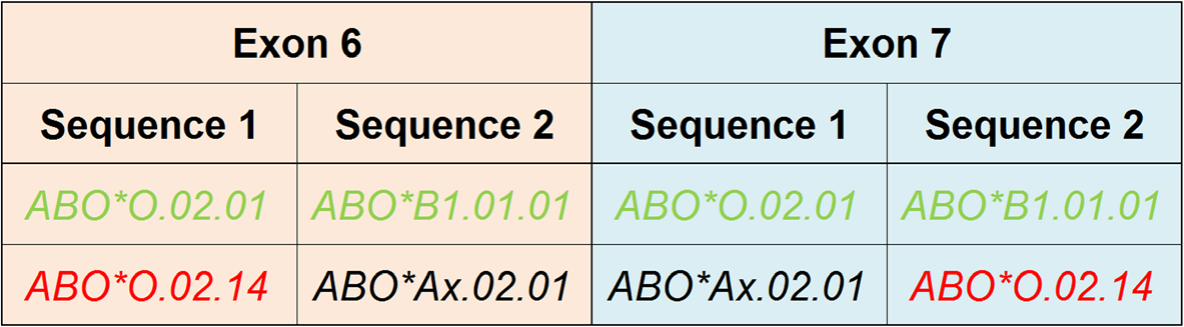
Phasing issues: Due to lack of phasing information between exons 6 and 7, the genotype ABO*O.02.01/ABO*B1.01.01 cannot be distinguished from the genotype ABO*O.02.14/ABO*Ax.02.01. For exon 6, sequence 1 may be explained by the alleles ABO*O.02.01 or ABO*O.02.14 (among others). The allele ABO*O.02.01 is composed of exon 6 sequence 1 and exon 7 sequence 1. The allele ABO*O.02.14 is composed of exon 6 sequence 1 and exon 7 sequence 2. Therefore, depending on the phasing between the exonic sequences, different typing results are possible: the rare Swedish *A* allele ABO*Ax.02.01 (black) in combination with an *O* allele (ABO*O.02.14) of unknown frequency (red) or the common *B* allele ABO*B1.01.01 and common *O* allele ABO*O.02.01 (both green)

In contrast to Sanger sequencing, NGS delivers phased information: Every read carries information about the phasing between two or more heterozygous positions. However, in a short amplicon sequencing workflow, phasing information between the individual amplicons is lacking. Often there is only one possibility for joining amplicons that results in valid sequence combinations of e.g. exon 6 and 7. Sometimes, however, both sequences of one exon can be joined with both sequences of the other exon. In such cases, the true sequences or alleles cannot be determined without additional information. For instance, analysis of all *ABO* alleles and their sequences showed that samples with the genotype ABO*O.02.01/ABO*B1.01.01 cannot be distinguished from the genotype ABO*O.02.14/ABO*Ax.02.01 due to the lack of phasing information between exons 6 and 7 (Fig. 2). Given the high abundance of the ABO*O.02.01 and ABO*B1.01.01 alleles and the low likelihood of a genotype combining the two rare alleles ABO*O.02.14 and ABO*Ax.02.01, we decided to disregard the possibility of an ABO*O.02.14/ABO*Ax.02.01 combination. Likewise the genotype *A1.01.02*/*O.02.14* is neglected in favor of the more likely *B1.01.01*/*O.02.02* allele combination. However, those rare alleles are unquestionably identified in all other genotypes.

### Validation

We validated our workflow by genotyping 468 samples with known serological blood group status. For 15 samples (3.2 %) typing failed, either because read counts in one of the amplicons were too low (8 samples), or because the allele groups identified in the 3 amplicons did not intersect and could therefore not be resolved unambiguously (7 samples).

451 (99.6 %) of the 453 successfully typed samples were concordant with the serological status. For 2 samples (0.4 %) genotyping results (*AO*) deviated from the serological status (O). Independent serological testing of fresh samples confirmed the O-type status for both individuals. On the other hand, the *AO* genotype obtained by sequencing was confirmed by an SSP assay. These conflicting results may partly be explained by the identified alleles: ABO*Ax.13.01.1 (sample 324) is expected to give rise to a very weak A phenotype [1, 15]. In accordance with that, the serum assay failed to detect anti-A2 antibodies at room temperature. The serological status for the other sample (226), however, did not show any abnormalities. The molecular genotype group includes several weak *A* alleles (ABO*Ael.04.01.1, ABO*Aw.13.01.1, ABO*Aw.15.01.1 and ABO*Ax.15.01.1) whose presence could explain the discrepant findings. Those alleles cannot be excluded by our approach as they differ from ABO*A1.01 only in intron 6 (ABO*Ael.04.01.1, ABO*Aw.15.01.1), in exon 1 (ABO*Aw.13.01.1) or in our primer binding site covering 20 positions in the 5’ region of exon 7 (ABO*Ax.15.01.1).

This demonstrates the limitations of an exon 6/7 restricted genotyping assay. Such a restricted assay can therefore currently not replace serological analysis. It does serve, though, as a cost effective extension that adds detailed allelic information. Conceptually, an addition of the missing exons (and introns) is straightforward. Within the scope of our approach, however, the *ABO* genotypes are supposed to serve as additional selection criteria within the search for HLA-matched unrelated hematopoietic stem cell donors. Furthermore, the ABO status provided by our screening approach has to be confirmed by standard methods during donor clearing before stem cell transplantation. Against this background, a deviation rate of 0.5 % from the serological status seems acceptable to us.

### ABO allele frequencies

The primary purpose of this project was to supplement HLA genotyping with basic ABO blood group information at low cost. However, the data generated lends itself to an allele-level frequency analysis. We chose to analyze a subset of 113,367 samples from individuals of German descent processed from June 2014 to September 2014. Low DNA concentration was identified as main source of error when working with PCR volumes in the sub-microliter range [16]. Therefore, the data set was restricted to samples with a DNA concentration of higher than 20 ng/μl in order to minimize the risk for wrong assignments which could distort the frequency calculations particularly of low frequency alleles.

As discussed above, two conceptual limitations interfere with full allele-level resolution for every sample: limited sequence coverage and missing phasing information. Despite the limited sequence coverage most observed alleles can be resolved to the third field (e.g. *A1.02.01*). However, three *A*, one *B* and five *O* genotypes include alleles spanning several subtypes (e.g. *A1* and *Aw*, *O.02* and *O.67*) (see Table 1 for the allele groups with ambiguities observed in the data set and Additional file 1: Table S3 for an exhaustive list of allele groups with ambiguities). Based on the remarks on the abundance of alleles and allele groups in “The Blood Group Antigen FactsBook” [17] we assume that most of those alternative alleles occur at low frequencies in the German population. However, given the large sample size, some of those alternative alleles may be present in the data set. This may result in a slight overestimation of the more abundant subgroups (e.g. A1, A2, B1) and an underestimation of the less frequent subgroups (e.g. Ax, Aw, Bw).

**Table 1:** Definition of allele groups spanning multiple ABO subgroups that cannot be resolved based on exons 6 and 7 and their constituent alleles

Allele group identifier	Allele	Blood group	Subgroup	German^a^	Caucasian^a^	Frequency	StDev
A1.01.01*	ABO*A1.01.01	A	A1	Common	Common	20.286 %	0.243 %
	ABO*A1.09.01.1	A	A1	Undefined	Undefined		
	ABO*Ael.04.01.1	A	Ael	Unknown	Unknown		
	ABO*Aw.13.01.1	A	Aw	Unknown	Present		
	ABO*Aw.15.01.1	A	Aw	Unknown	Unknown		
	ABO*Aw.29.01.1	A	Aw	Unknown	Present		
	ABO*Ax.15.01.1	A	Ax	Unknown	Unknown		
O.02.01*	ABO*O.02.01	O	O2	Many	Many	18.479 %	0.222 %
	ABO*O.02.17.1	O	O2	Undefined	Undefined		
	ABO*O.67.01	O	O67	Undefined	Undefined		
B1.01.01*	ABO*B1.01.01	B	B1	Common	Common	8.420 %	0.113 %
	ABO*Bw.new	B	Bw	Undefined	Undefined		
A2.01.01*	ABO*A2.01.01	A	A2	Common	Common	6.588 %	0.088 %
	ABO*A2.01.02.1	A	A2	Common	Common		
	ABO*A2.16.01.1	A	A2	Unknown	Unknown		
	ABO*Aw.09.01.1	A	Aw	Unknown	Unknown		
	ABO*Aw.16.01.1	A	Aw	Unknown	Unknown		
	ABO*Aw.17.01.1	A	Aw	Unknown	Present		
	ABO*Aw.22.01.1	A	Aw	Unknown	Unknown		
	ABO*Aw.27.01.1	A	Aw	Unknown	Unknown		
O.02.15*	ABO*O.02.15.1	O	O2	Undefined	Undefined	1.610 %	0.032 %
	ABO*O.02.18.1	O	O2	Undefined	Undefined		
O.02.02*	ABO*O.02.02	O	O2	Unknown	Unknown	0.137 %	0.008 %
	ABO*O.02.20.1	O	O2	Undefined	Undefined		
	ABO*O.54.01.1	O	O54	Undefined	Undefined		
A2.12.01*	ABO*A2.09.01.1	A	A2	Unknown	Unknown	0.043 %	0.004 %
	ABO*A2.12.01.1	A	A2	Unknown	Unknown		
	ABO*Aw.26.01.1	A	Aw	Unknown	Unknown		
O.01.11*	ABO*O.01.11.1	O	O1	Undefined	Undefined	0.014 %	0.003 %
	ABO*O.06.01.1	O	O6	Unknown	Rare		
O.02.07*	ABO*O.02.07.1	O	O2	Undefined	Undefined	0.008 %	0.002 %
	ABO*O.40.01.1	O	O40	Undefined	Undefined		

^a^Allele frequency classifications after [15]

*Allele groups as defined here are marked by an asterisk throughout the text

Depending on the alleles present in a particular sample, the missing phase information may create an additional level of ambiguity for genotyping. However, when analyzing a large data set retrospectively, a Bayesian probabilistic framework allows to partially account for the uncertainty in the data. In short, the algorithm takes advantage of the fact that the frequencies can be determined for many samples without phasing problem. Based on the frequencies for problem-free samples, the frequencies for the samples with phasing problems are estimated. In addition, we obtain an estimate of the level of uncertainty for each frequency estimate.

**Fig. 3:**
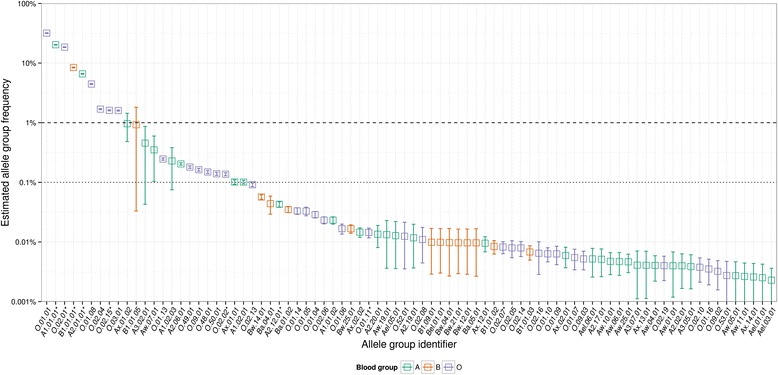
Bayesian *ABO* allele and allele group frequency estimates for 113,367 German samples. Error bars denote standard deviations around the posterior mean based on 10,000 MCMC iterations. Blood group phenotypes are color-coded in blue (A), red (B) and purple (O). The constituent alleles combined under allele group identifiers are provided in Table 1. Allele group identifiers ending with an asterisk combine alleles across subgroups

When applied to our data set of 113,367 genotyped samples this approach delivered frequency estimates for 82 alleles or allele groups (Fig. 3 and Table 2) ranging from 0.002 % to 32 %. To our knowledge this is the first estimation of allelic *ABO* frequencies on such a large dataset.

**Table 2:** Population-wide frequency estimates for blood groups, subgroups and allele groups

A	29.482 %	B	9.570 %	O	60.948 %
A1	20.638 %	B1	9.369 %	O1	36.619 %
A1.01.01*	20.286 %	B1.01.01*	8.420 %	*O.01.01*	31.772 %
*A1.01.02*	0.023 %	*B1.01.02*	0.008 %	*O.01.04*	0.029 %
*A1.02.01*	0.101 %	*B1.01.03*	0.007 %	*O.01.05*	0.033 %
*A1.02.03*	0.228 %	*B1.01.05*	0.924 %	*O.01.06*	0.017 %
A2	6.869 %	*B1.09.01*	0.010 %	*O.01.07*	0.005 %
A2.01.01*	6.588 %	Ba	0.089 %	*O.01.08*	4.453 %
*A2.02.01*	0.004 %	*Ba.01.02*	0.035 %	*O.01.09*	0.006 %
*A2.06.01*	0.204 %	*Ba.04.01*	0.044 %	*O.01.10*	0.006 %
A2.12.01*	0.043 %	*Ba.05.01*	0.010 %	O.01.11*	0.014 %
*A2.17.01*	0.005 %	Bel	0.010 %	*O.01.13*	0.246 %
*A2.19.01*	0.012 %	*Bel.01.01*	0.010 %	*O.01.14*	0.033 %
*A2.20.01*	0.013 %	Bw	0.103 %	*O.01.16*	0.004 %
A3	0.462 %	*Bw.04.01*	0.010 %	O2	22.079 %
*A3.02.01*	0.454 %	*Bw.12.01*	0.010 %	O.02.01*	18.479 %
*A3.05.01*	0.004 %	*Bw.14.01*	0.057 %	O.02.02*	0.137 %
*A3.07.01*	0.004 %	*Bw.21.01*	0.010 %	*O.02.04*	1.689 %
Ael	0.023 %	*Bw.25.01*	0.017 %	*O.02.05*	0.008 %
*Ael.01.01*	0.003 %			*O.02.06*	0.023 %
*Ael.03.01*	0.002 %			O.02.07*	0.008 %
*Ael.05.01*	0.013 %			*O.02.08*	0.011 %
*Ael.06.01*	0.005 %			*O.02.10*	0.004 %
Aw	0.391 %			*O.02.13*	0.091 %
*Aw.01.01*	0.004 %			*O.02.14*	0.008 %
*Aw.04.01*	0.004 %			O.02.15*	1.610 %
*Aw.05.01*	0.003 %			*O.02.16*	0.006 %
*Aw.06.01*	0.005 %			*O.02.19*	0.004 %
*Aw.07.01*	0.351 %			O3	1.597 %
*Aw.10.01*	0.005 %			*O.03.01*	1.597 %
*Aw.11.01*	0.003 %			O48	0.149 %
*Aw.19.01*	0.013 %			*O.48.01*	0.149 %
*Aw.25.01*	0.005 %			O49	0.180 %
Ax	1.099 %			*O.49.01*	0.180 %
*Ax.01.01*	0.101 %			O50	0.139 %
*Ax.01.02*	0.961 %			*O.50.01*	0.139 %
*Ax.02.01*	0.006 %			O52	0.012 %
*Ax.02.02*	0.015 %			*O.52.01*	0.012 %
*Ax.12.01*	0.010 %			O53	0.003 %
*Ax.13.01*	0.004 %			*O.53.01*	0.003 %
*Ax.14.01*	0.003 %			O9	0.171 %
				*O.09.01*	0.163 %
				*O.09.02*	0.003 %
				*O.09.03*	0.005 %

*Allele groups as defined here are marked by an asterisk throughout the text

### High-resolution genotyping

We further explored the possibilities for high-resolution genotyping based on exon 6 and 7 sequencing. While an algorithm as discussed in the previous paragraph is limited to retrospective frequency estimations, these estimates may help distinguishing between alternative allele combinations based on their relative likelihood. Given sufficient difference, the less likely allele combination may be ignored accepting a minor increase in the error rate. In cases of allele combinations with similar likelihood both allele combinations should be included in the genotype result reducing the resolution.

We analyzed all observed genotyping results with unresolved phasing with regard to their frequencies and the relative likelihoods of their allele combinations (Table 3). We identified 24 unique cases with diverse properties in our data set. The 15 least abundant cases have a cumulative frequency of 0.11 % and will affect only very few samples. The six most abundant cases sum up to a cumulative frequency of 32 %, demonstrating the prevalence of the issue. Likewise the relative likelihoods of two possible allele combinations range from close to 1 to several millions. Two cases (case 4 and 11, Table 3) as discussed above would lead to a different *ABO* genotype (*OB1* versus *OAx*/*OA1*). Disregarding the *OAx*/*OA1* combination seems warranted without an inadequate error risk at least for German samples: Even though the *OB1*/*OA1* ambiguity has only an odds ratio of 6,271, given the low frequency of that ambiguity this would theoretically result in one error in 23 million typed samples. Twelve of the remaining cases are irrelevant for determining the subgroups as the ambiguities affect only the next field (e.g. A2.01.01* versus *A2.06.01*, *O.01.01* versus *O.01.05*). In most circumstances those differences will not be of interest. Two cases remain with a frequency above 0.1 % and an effect on subgroup results: For case 2 the *OAx* combination can be safely ignored, having a relative likelihood of smaller than 1/100,000. Case 3 remains unpleasant as it combines a high frequency of 4.5 % with a moderately low relative likelihood of one in 617. In most scenarios an error rate of 1 in 10,000 samples (4.5 % divided by 617) is probably acceptable. Otherwise in 4.5 % of the samples *OA2* versus *OA1* cannot be resolved. We conclude that the missing phasing information does not interfere with high-resolution genotyping for German samples as for 99.9 % of the samples phasing could be resolved at least to the subgroup level if an error rate of 0.01 % (*A1* versus *A2*, case 3) is acceptable.

**Table 3:** Genotypes and their corresponding frequencies where the lack of phasing information leads to alternative outcomes (Group A or B). The relative likelihood of A vs. B is estimated based on allele frequencies

Case #	Case	Allele group 1a		Allele group 2a		Allele group 1b		Allele group 2b		Relative likelihood	Subgroup relevance
	Frequency	Identifier	Frequency	Identifier	Frequency	Identifier	Frequency	Identifier	Frequency	a/b	
1	12.545 %	*O.01.01*	31.772 %	O.02.01*	18.479 %	O.01.11*	0.014 %	O.02.02*	0.137 %	298,326	No
2	8.092 %	A1.01.01*	20.286 %	O.02.01*	18.479 %	*Ax.02.02*	0.015 %	O.02.02*	0.137 %	187,949	Yes
3	4.511 %	A2.01.01*	6.588 %	*O.01.01*	31.772 %	A1.01.01*	20.286 %	*O.01.06*	0.017 %	617	Yes
4	3.250 %	B1.01.01*	8.420 %	O.02.01*	18.479 %	*Ax.02.01*	0.006 %	*O.02.14*	0.008 %	3,313,940	Yes
5	2.868 %	A1.01.01*	20.286 %	A2.01.01*	6.588 %	*A1.02.01*	0.101 %	*A2.06.01*	0.204 %	6,501	No
6	1.095 %	*O.01.01*	31.772 %	O.02.15*	1.610 %	*O.01.14*	0.033 %	*O.02.05*	0.008 %	193,859	No
7	0.113 %	*O.01.01*	31.772 %	*O.09.01*	0.163 %	*O.01.05*	0.033 %	*O.09.03*	0.005 %	30,220	No
8	0.071 %	*A1.02.01*	0.101 %	*O.01.01*	31.772 %	A1.01.01*	20.286 %	*O.01.05*	0.033 %	5	No
9	0.068 %	A1.01.01*	20.286 %	*O.09.01*	0.163 %	*A1.02.01*	0.101 %	*O.09.03*	0.005 %	6,284	No
10	0.027 %	A2.01.01*	6.588 %	*O.01.05*	0.033 %	*A1.02.01*	0.101 %	*O.01.06*	0.017 %	128	Yes
11	0.026 %	B1.01.01*	8.420 %	O.02.02*	0.137 %	*A1.01.02*	0.023 %	*O.02.14*	0.008 %	6,271	Yes
12	0.016 %	*O.01.01*	31.772 %	*O.02.06*	0.023 %	*O.01.10*	0.006 %	O.02.02*	0.137 %	853	No
13	0.010 %	*A1.01.02*	0.023 %	O.02.01*	18.479 %	*Ax.02.01*	0.006 %	O.02.02*	0.137 %	528	Yes
14	0.007 %	*Ax.02.02*	0.015 %	*O.01.01*	31.772 %	A1.01.01*	20.286 %	O.01.11*	0.014 %	2	Yes
15	0.005 %	*A1.02.01*	0.101 %	O.02.15*	1.610 %	*A2.20.01*	0.013 %	*O.02.05*	0.008 %	1,515	Yes
16	0.003 %	*O.01.10*	0.006 %	O.02.01*	18.479 %	O.01.11*	0.014 %	*O.02.06*	0.023 %	350	No
17	0.002 %	A1.01.01*	20.286 %	*Ax.02.01*	0.006 %	*A1.01.02*	0.023 %	*Ax.02.02*	0.015 %	356	No
18	0.002 %	A1.01.01*	20.286 %	*Aw.25.01*	0.005 %	*A1.02.01*	0.101 %	*A3.02.01*	0.454 %	2	Yes
19	0.002 %	A1.01.01*	20.286 %	*B1.01.02*	0.008 %	*A1.01.02*	0.023 %	*B1.01.05*	0.924 %	8	No
20	0.002 %	*Aw.25.01*	0.005 %	*O.01.01*	31.772 %	*A3.02.01*	0.454 %	*O.01.05*	0.033 %	10	Yes
21	0.001 %	*A1.01.02*	0.023 %	A2.01.01*	6.588 %	*A1.02.03*	0.228 %	*A2.06.01*	0.204 %	3	No
22	0.001 %	A2.01.01*	6.588 %	O.01.11*	0.014 %	*Ax.02.02*	0.015 %	*O.01.06*	0.017 %	389	Yes
23	0.001 %	A1.01.01*	20.286 %	*Aw.01.01*	0.004 %	*A2.06.01*	0.204 %	*A2.17.01*	0.005 %	78	Yes
24	0.001 %	A2.01.01*	6.588 %	*O.09.03*	0.005 %	*A2.06.01*	0.204 %	*O.09.01*	0.163 %	1	No

*Allele groups as defined here are marked by an asterisk throughout the text

### Novel alleles

An intrinsic advantage of sequencing approaches compared to other molecular methods is the ability to identify and characterize novel alleles. As of March 2016 we have genotyped 1,693,287 samples for *ABO*. We identified 20,190 samples (1.2 %) with indications for the presence of a novel allele. As the characterization of novel alleles was not the primary focus of the project, we attempted verification by replicate sequencing only for a subset of 4,375 samples (21.7 %). That subset was not systematically selected over the time course of the project. It rather reflects historic changes in analysis policies and automation capacities. For 815 samples (18.6 %) the replicate sequencing confirmed the presence of a novel allele with the identical so far unreported sequence. A total of 287 unique novel allelic sequences were found (Additional file 1: Table S4). While 193 of these sequences were encountered only once, the ten most common sequences were identified in 369 different samples. The most abundant sequence was identified in 174 samples correlating to a frequency of 0.01 % in our data set. Based on the confirmation rate of 18.6 % for the reanalyzed samples, we estimate that about 3,761 of the 20,190 originally flagged samples contain novel alleles. That would correlate to an overall frequency of novel alleles of 0.22 %. To exclude the unlikely possibility of systematic errors due to the applied technology we analyzed a random selection of 37 samples representing 26 unique novel allelic sequences a third time by PacBio sequencing (Additional file 1: Table S4), an orthogonal technology with completely different error profile. All PacBio resequencing results confirmed the original findings. This study demonstrates the potential of our workflow for the detection of novel alleles. To submit the identified novel sequences to the BGMUT database more work is required. In particular an approach that covers the whole gene and provides fully phased sequence information should be applied. Based on our experience with the submission of fully phased HLA genes we intend to embark on this task soon.

## Discussion

Here, we propose a cost-effective workflow for high-throughput *ABO* genotyping. Despite the restricted coverage of exons 6 and 7, the approach delivers allelic or allele group level resolution for 99.9 % of the samples. While the frequency analysis based on 113,367 samples from individuals of German descent allowed us to propose ways to handle ambiguities originating from unresolved phasing, we lack such detailed frequency information for the alleles compromising the not resolvable allele groups (Table 1) whose sequences differ only in the regions not covered by our approach. Therefore we cannot judge if those alleles prevent *ABO* subgroup-level resolution since they may appear too frequent to disregard them. This limitation is however shared with many published molecular approaches that *a priori* limit the data basis to the more frequent alleles that are readily distinguishable. Our workflow, however, lends itself to extend the targeted region to the other exons. Such an extended workflow could deliver true allelic level *ABO* genotyping and resolve the frequencies of the less common alleles in the so far unresolved allele groups. Even such an extended workflow would still be very cost-effective. Main cost factors of our workflow, when applied at high throughput, are DNA isolation, PCR reactions (including target-specific and barcoding primer oligonucleotides) and sequencing. Costs for DNA isolation and PCR reactions depend largely on the chosen reagent providers. Current reagent costs for sequencing on an Illumina MiSeq (2x300 bp) are well below 1 € per 20,000 reads which would deliver more than plenty of reads to cover the whole *ABO* gene. This assumes, however, that *ABO* genotyping is performed together with other targets and/or that the throughput is sufficiently high to utilize the full capacity of the instrument. In our setup, *ABO* genotyping is performed alongside of genotyping of six HLA loci, *CCR5Δ32*, *KIR* and the *Rh* blood group. Up to 4,800 samples are jointly analyzed on one rapid-run flow-cell on HiSeq 2500 instruments resulting in 60,000 reads per sample on average, at sequencing reagent costs of about 1 €. This underscores the cost effectiveness of the described workflow. Given the low sequencing costs, applying these strategies to an extended blood group panel seems feasible. The major challenge would lie in developing highly multiplexed efficient PCR assays targeting the genes of interest.

While the workflow is slim compared to other sequencing approaches, the sequencing alone runs for two full days. Taken together, genotyping results can be obtained within four days. However, in a high-throughput optimized setting the turn-around-time would probably extend to two or three weeks.

## Conclusions

The application of next generation sequencing to blood group genotyping has been proposed [9, 10] and the feasibility demonstrated in proof-of-concept studies [18–20]. We report the application of NGS to ABO analysis and successfully genotyped more than 1.5 million samples. Despite the restricted focus on exons 6 and 7 the data enabled us to report frequency data on 82 distinguished alleles or allele groups. For most of the less abundant alleles this constitutes the first quantitative frequency estimation. While this approach can by no means substitute serological ABO status analysis, it could serve as a cost-effective complementation to reveal the molecular *ABO* genotype.

## Methods

### Samples, DNA isolation and quantification

Samples were provided by DKMS German Bone Marrow Donor Center and other donor centers for HLA and blood group typing between April 2014 and December 2015. DNA was isolated from 150 μl whole blood or a single buccal swab using the magnetic-bead-based “chemagic DNA Blood Kit special” or “chemagic DNA Buccal Swab kit special” (Perkin Elmer, Baesweiler, Germany), respectively. DNA was eluted in 100 μl elution buffer (10 mM Tris-HCl pH8.0). DNA concentrations were measured by fluorescence (SYBR Green, Biozym, Hessisch Oldendorf, Germany) using the TECAN infinite 200Pro (Tecan, Männedorf, Switzerland) plate reader. Samples with concentrations of less than 2 ng/μl were excluded from ABO typing.

### PCR amplification

PCR amplification was performed using 48.48 or 192.24 Fluidigm Access Array IFCs (Fluidigm Corporation, South San Francisco, USA) in combination with the Roche High Fidelity Fast Start Kit (Roche, Basel, Switzerland) as previously described [14]. We used a thermal profile of 50 °C for 2 min, 70 °C for 20 min, 95 °C for 10 min, followed by 20 cycles at 95 °C for 25 s, 60 °C for 30 s and 72 °C for 90 s and additional 15 cycles at 95 °C for 25 s, 50 °C for 30 s and 72 °C for 90 s and a finishing step at 72 °C for 5 min. PCR setup included the target-specific and the barcoding primers.

Alternatively, amplification was performed in 384-well plates with 2 μl template DNA, 1 μl 10x buffer mix without MgCl_2_ (Roche Fast Start Kit), 0.8 μl 25 mM MgCl_2_, 0.5 μl DMSO, 0.2 μl 10 mM dNTPs each (Roche Fast Start Kit), 0.1 μl Fast Start Taq Polymerase (5 U/μl) (Roche Fast Start Kit), 4.4 μl PCR grade water and 1 μl of target-specific primer mix. We used a thermal profile of 95 °C for 4 min followed by 35 cycles at 95 °C for 25 s, 57 °C for 30 s and 72 °C for 90 s, and a finishing step at 72 °C for 5 min. Amplicons belonging to one sample were pooled with an CyBi-Well Vario system (Analytik Jena AG, Jena, Germany) and 2 μl transferred to an 9 μl pre-aliquoted PCR master mix including 1 μl 10x buffer mix without MgCl_2_ (Roche Fast Start Kit), 1 μl 25 mM MgCl_2_, 0.2 μl DMSO, 0.2 μl 10 mM dNTPs each (Roche Fast Start Kit), 0.1 μl Fast Start Taq Polymerase (5 U/μl) (Roche Fast Start Kit), 3.5 μl PCR grade water as well as 2 μl of barcode primers (2 μM equimolar mix of index 1 and index 2). Barcoding PCR was performed with the following thermal profile: 95 °C for 4 min, 10 cycles at 95 °C for 25 s, 50 °C for 30 s and 72 °C for 90 s, and a finishing step at 72 °C for 5 min.

We employed two different types of assays (ABO assay 1 and ABO assay 2, compare Fig. 1). For ABO assay 1, PCR was performed for three amplicons, exon 6 (192 bp), exon 7 amplicon a (460 bp) and exon 7 amplicon b (328 bp). Amplification reactions for exon 6 (0.85 μM each primer) and exon 7a (0.75 μM each primer) were multiplexed, amplification for exon 7b (0.5 μM each primer) was performed separately. For ABO assay 2, only the central portion of exon 7 (507 bp) was amplified and amplification reactions for exon 6 (0.3 μM each primer) and exon 7 (0.25 μM each primer) were multiplexed.

Target-specific primers and index primers were obtained from metabion (metabion international AG, Planegg, Germany).

### Library preparation and sequencing

For sequencing library preparation we pooled 48 barcoded samples from a 48.48 Access Array, 2 x 96 barcoded samples from a 192.24 Access Array, or all barcoded amplicons from one 384-well plate, respectively.

Pooled PCR products were purified with SPRIselect Beads (BeckmanCoulter, Brea, USA) using a ratio of 0.7:1 beads to PCR product. Purified amplicon pools were diluted 1:4000 for quantification by qPCR. Pooling, purification and subsequent dilution for qPCR quantification were performed on Biomek 3000 or Biomek NX workstations (Beckman Coulter, Brea, USA). qPCR was performed on an ABI-StepOnePlus qPCR cycler (Thermo Fisher, Carlsbad, USA) using the Library Quant Illumina Kit (KAPA Biosystems, Boston, USA) with standards in a range from 0.2 fM to 20 pM.

The purified and quantified amplicon pools were mixed in equimolar amounts and prepared as recommended by Illumina (MiSeq Reagent Kit v2-Reagent Preparation Guide). Libraries were loaded at 12.5 to 18.5 pM onto MiSeq or HiSeq flow cells with 10 % PhiX spiked in. Paired-end sequencing was performed at 249, 251 or 260 (ABO assay 2) cycles.

Confirmatory sequencing of novel *ABO* alleles was performed using single molecule real-time (SMRT) sequencing on a Pacific Bioscience RS II instrument. *ABO* genes spanning exons 3 to 7 were amplified by long-range PCR. A barcoded sequencing library was prepared using the SMRTbell Template Prep Kit 1.0 (Part number 100-259-100) from Pacific Biosciences following standard protocols. Base calling and demultiplexing was performed using Pacific Biosciences’ SMRT Portal. Sequence reads were mapped against *ABO* reference alleles (ABO*A1.01.01.1 for antigens A and O, ABO*B1.01.01.1 for antigen B) with BWA-MEM [21] using PacBio settings (-x pacbio) and subsequently visually inspected in IGV [22].

### Genotyping

Next generation sequencing based *ABO* genotyping was implemented in the genotyping application neXtype using the same principles as described previously for HLA allele typing [13]. Briefly, neXtype utilizes a set of reference allele sequences against which query sequences are matched for each exon separately. If different alleles share an exonic sequence, a query sequence will match multiple target alleles per exon. We refer to these sets of matched target alleles as Exon Allele Groups (EAGs). A final *ABO* genotype assignment for a sample is obtained by intersecting the member alleles of all EAGs across exons 6, 7a and 7b. If only one (in a homozygous sample) or two (in a heterozygous sample) alleles are shared across EAGs across exons, the sample *ABO* genotype can be fully resolved. If multiple alleles share the same sequences in the amplified region, those alleles cannot be resolved unambiguously. We distinguish the following levels of resolution (low to high): blood group (A, B, O), subgroup (e.g. A1, Ax, B1, O1, O2), allele groups (set of alleles derived from at least two subgroups), second field allelic resolution (e.g. *A1.01*), third field allelic resolution (e.g. *A1.01.02*) and full allelic resolution (e.g. *A1.01.01.1*). Allele group identifiers and their constituent alleles are provided in Table 1.

An additional layer of ambiguity arises if, e.g., the two EAGs at one exon intersect each with two EAGs at another exon. In this case the phase between exons cannot be resolved and multiple solutions for the underlying genotype exist.

As a reference for *ABO* typing by neXtype we used the 280 *ABO* alleles currently available from the NCBI dbRBC Alignment Viewer (http://www.ncbi.nlm.nih.gov/projects/gv/rbc/align.fcgi?cmd=aligndisplay&user_id=0&probe_id=0&source_id=0&locus_id=61&locus_group=3&proto_id=0&kit_id=0&banner=1#). Seven *ABO* alleles lacked exon 6/7 sequence information, in four alleles the major part of exon 7 is unknown, 19 alleles lacked phenotype information, and for one allele the phenotype is uncertain (Additional file 1: Table S2). These alleles were excluded, reducing our reference allele set to 108 *A*, 68 *B* and 73 *O* alleles.

### Allele group frequency estimation

For *ABO* allele group frequency estimation a subset of 124,206 donor samples typed between June 2014 and September 2014 for DKMS German Bone Marrow Donor Center was extracted from the total data set. Only samples with a DNA concentration ≥ 20 ng/μl were included. For each sample, genotyping was performed using neXtype as described above. 123,250 of the samples could be successfully genotyped. Therefore, the failure rate for these high quality samples was below 1 %. Based on self-declared ethnic origin, 113,367 of these donors were of German descent and therefore included in the final analysis. For 67 % of these samples (76,276) an unambiguous genotype or allele group genotype could be assigned, i.e. there was no phasing ambiguity across exons (see above). In 33 % of the cases (37,091 samples) multiple genotypes or allele group genotypes could be mapped to the particular EAG compositions. Both groups were included in further analyses.

To estimate allele frequencies, we mapped observed genotype frequencies for unambiguous genotype assignments to allele frequencies assuming standard Hardy-Weinberg proportions. For ambiguous genotype assignments, observed frequencies were mapped to the sum of the individual genotype probabilities that an ambiguous genotype was composed of. We used these mappings to formulate a Bayesian model for multinomial frequency estimation implemented in JAGS (version 3.4.0, http://mcmc-jags.sourceforge.net) [23]. Additional file 2 (ABO_model.bug) provides the model specifications in the BUGS language [24]. Additional file 3 (ABO_count.data) provides the raw count data for EAG groups in the order in which they appear in the model. Additional file 4 (AlleleGroupIdentifiers.csv) provides the mapping from EAG group codes to the allele group identifiers used in this paper. The mean and standard deviation of allele group frequency estimates were estimated from the posterior distribution generated by 10,000 MCMC iterations after a burn-in of 10,000 iterations. All calculations were performed in R (version 3.2.2) [25], using the package rjags (version 3-15, http://CRAN.R-project.org/package=rjags).

### Validation

#### Serology

Serological characterization of the blood samples was performed by German Red Cross (DRK) Blood Donor Service Nord-Ost gGmbH (Institut Chemnitz) and DRK Blood Donor Service Baden-Württemberg - Hessen, Ulm and Baden-Baden, Germany.

#### SSP-PCR

The “RBC-Ready Gene ABO” SSP-PCR-Kit (Inno-Train Diagnostik GmbH, Kronberg im Taunus, Germany) was used according to manufacturer’s instructions.

## Additional files

Additional file 1: Table S1. Primer sequences for ABO assay 1 and 2. **Table S2.** Alleles excluded from the reference allele set downloaded from NCBI dbRBC. **Table S3.** Alleles included in our final reference allele set and definition of allele groups as used in this study. Prevalence information of alleles are based on “*The Blood Group Antigen FactsBook”* [17], and the estimated frequencies are based on 113,367 German samples. **Table S4.** Alleles identified with so far undescribed nucleotide substitutions and structural variations. We provide numbers of observations per allele and positions of nucleotide changes relative to the cDNA of the reference alleles (*ABO*A1.01.01.1* for antigens A and O, *ABO*B1.01.01.1* for antigen B). A putative antigen designation was derived from sequence similarities to the exons harboring the nucleotide changes. (XLSX 38 kb) Additional file 2: JAGS model specification file (ABO_model.bug). (BUG 38 kb) Additional file 3: EAG group frequency counts (ABO_count.data) as input for the JAGS model specified in “ABO_model.bug”. (DATA 879 bytes) Additional file 4: Mappings from EAG group codes as used in the JAGS model calculations to the allele groups (AlleleGroupIdentifiers.csv). (CSV 6 kb)
